# Complete Genome Sequence of a Human-Invasive *Salmonella enterica* Serovar Typhimurium Strain of the Emerging Sequence Type 213 Harboring a Multidrug Resistance IncA/C Plasmid and a *bla*_CMY-2_-Carrying IncF Plasmid

**DOI:** 10.1128/genomeA.01323-15

**Published:** 2015-11-12

**Authors:** Claudia Silva, Edmundo Calva, Juan J. Calva, Magdalena Wiesner, Marcos Fernández-Mora, José L. Puente, Pablo Vinuesa

**Affiliations:** a Departamento de Microbiología Molecular, Instituto de Biotecnología, Universidad Nacional Autónoma de México, Cuernavaca, Morelos, Mexico; b Department of Infectious Diseases, Instituto Nacional de Ciências Médicas y Nutrición Salvador Zubirán, Mexico City, Mexico; c Centro de Ciencias Genómicas, Programa de Ingeniería Genómica, Universidad Nacional Autónoma de México, Cuernavaca, Morelos, Mexico

## Abstract

*Salmonella enterica* subsp. *enterica* serovar Typhimurium strain 33676 was isolated in Mexico City, Mexico, from a patient with a systemic infection, and its complete genome sequence was determined using PacBio single-molecule real-time technology. Strain 33676 harbors an IncF plasmid carrying the extended-spectrum cephalosporin gene *bla*_CMY-2_ and a multidrug resistance IncA/C plasmid.

## GENOME ANNOUNCEMENT

Here, the complete genome sequence of *Salmonella enterica* subsp. *enterica* serovar Typhimurium strain 33676 is presented. It was isolated in 2011 in Mexico City, Mexico, from a blood culture taken from a 15-year-old woman suffering a febrile illness with invasive severe pancolitis, which was refractory to therapy with extended-spectrum cephalosporins (M. Wiesner, J. J. Calva, V. H. Bustamante, D. Pérez-Morales, M. Fernández-Mora, E. Calva, and C. Silva, unpublished data). Strain 33676 was assigned to sequence type 213 (ST213), which is the predominant genotype recovered in Mexico since 2001 (1, 2), and it is multidrug resistant, expresses FljB H2-phase flagella, and displays attenuated virulence on mice (Wiesner et al., unpublished data).

Genomic DNA was extracted by standard protocols (3) and sheared into ~10-kb fragments for PacBio library preparation and P5-C3 sequencing on two SMRT cells. The continuous long reads were assembled using the HGAP/Quiver-protocol in SMRT Portal version 2.3.0.140936.p4 (4), resulting in an assembly with 4 contigs. These were circularized by trimming the terminal repeats with Minimus2 (5) and subjected to two consecutive rounds of read remapping with the RS_Resequencing.1 module for sequence polishing, resulting in a final assembly with a mean coverage of ~119×. The size of the assembled genome is 5,088,186 bp, with a G+C content of 52.13%, comprising a 4.81-Mb chromosome and three plasmids: a multidrug resistance pIncA/C (161.5 kb), a pIncF carrying the *bla*_CMY-2_ gene (112.6 kb), and a small plasmid (4.5 kb).

Gene calling and annotation were performed with a modified version of Prokka (6). A total of 5,032 genes, 4,767 coding sequences (CDSs), and 30 pseudogenes were identified, which is ~4× more pseudogenes than those found (*n* = 7) in the previously sequenced ST213 genome of strain YU39 (1). Additionally, genes for 85 tRNAs, 22 rRNAs, and 1 transfer-messenger RNA (tmRNA) were annotated, plus 127 noncoding RNAs (ncRNAs), 3 clustered regularly interspaced short palindromic repeat (CRISPR) arrays, 3 riboswitches, and 444 signal peptides. The annotation was manually curated, adding prophage predictions made by the PHAST server (7), and genomic islands were detected by IslandViewer 3 (8).

Four complete prophages were located on the chromosome: ST104, Gifsy-2, ST64B, and Gifsy-1, plus several phage remnants. Two class 1 integrons were found on the pIncA/C plasmid that contribute to the multidrug-resistant phenotype: integron In27 (*dfrA12*, *gcuF*, and *aadA12*), which was previously reported for other ST213 strains (1, 2, 9), is widespread in *Enterobacteriaceae* plasmids; and In1003 (*estX-3*, *psp*, *aadA2*, and *qacH2*), which is reported for *Escherichia coli* plasmids in the INTEGRALL database (10), was detected in this study for the first time in *Salmonella*. A third resistance region on the pIncA/C plasmid contains the *floR*, *tetA*, *tetR*, *strB*, *strA*, and *sul2* genes linked to an IS*CR2* element (SE15cs_04884) (11). Additionally, several efflux pumps were identified, including the resistance-nodulation-division (RND) multidrug efflux pump (OqxAB) found in the YU39 pIncA/C and other *Enterobacteriaceae* plasmids (12). The extended-spectrum cephalosporinase gene *bla*_CMY-2_ was confirmed to be located on the pIncF plasmid. The orientation of the invertible H segment containing the promoter of the *fljBA* operon explains H2 phase flagellin expression (13).

### Nucleotide sequence accession numbers.

The complete sequences of the chromosome and the three plasmids of *Salmonella* Typhimurium strain 33676 are available in GenBank under accession numbers CP012681, CP012682, CP012683, and CP012684.
